# Long noncoding RNA DANCR is activated by SALL4 and promotes the proliferation and invasion of gastric cancer cells

**DOI:** 10.18632/oncotarget.23019

**Published:** 2017-12-06

**Authors:** Lei Pan, Wei Liang, Jianmei Gu, Xueyan Zang, Zhenhua Huang, Hui Shi, Jingyan Chen, Min Fu, Peng Zhang, Xiudi Xiao, Hui Qian, Wenrong Xu, Pengcheng Jiang, Xu Zhang

**Affiliations:** ^1^ Department of Breast Surgery, The Affiliated People's Hospital of Jiangsu University, Zhenjiang, Jiangsu 212002, China; ^2^ Jiangsu Key Laboratory of Medical Science and Laboratory Medicine, School of Medicine, Jiangsu University, Zhenjiang, Jiangsu 212013, China; ^3^ Department of Clinical Laboratory, Nantong Tumor Hospital, Nantong, Jiangsu 226361, China; ^4^ Institute of Digestive Diseases, The Affiliated People's Hospital of Jiangsu University, Zhenjiang, Jiangsu 212002, China; ^5^ Department of General Surgery, The Affiliated People's Hospital of Jiangsu University, Zhenjiang, Jiangsu 212002, China

**Keywords:** gastric cancer, lncRNA, DANCR, SALL4, progression

## Abstract

Long noncoding RNAs (LncRNAs) play important roles in tumor development and progression. The expression of lncRNAs is frequently dysregulated in human cancer. DANCR (anti-differentiation noncoding RNA) is a newly identified lncRNA in human cancer, however, its functional roles and clinical value in gastric cancer (GC) remains unknown. In this study, we investigated the expression of DANCR in the tumor tissues and serum of GC patients and analyzed the correlation between DANCR expression levels and the clinicopathological characteristics. Our results showed that the expression of DANCR was higher in the tumor tissues than that in the adjacent non-cancerous tissues. The expression level of DANCR was also elevated in the serum of GC patients compared to that of healthy controls. The expression levels of DANCR were significantly associated with tumor size, TNM stage, lymphatic metastasis and invasion depth. DANCR knockdown inhibited the proliferation of GC cells by inducing cell cycle arrest and cell apoptosis. In addition, DANCR knockdown suppressed gastric cancer growth *in vivo*. Moreover, DANCR knockdown inhibited the migration and invasion of GC cells via the suppression of epithelial-mesenchymal transition (EMT). However, DANCR overexpression had the opposite effect. DANCR is activated by SALL4 in gastric cancer cells and exerted its oncogenic activities through the activation of β-catenin pathway. Taken together, our findings suggest that DANCR promotes the progression of gastric cancer and have the potential to serve as a novel diagnostic biomarker.

## INTRODUCTION

Gastric cancer (GC) is the third leading cause of cancer-related death worldwide [1, 2]. The occurrence of GC is a complex process that involves multiple genes including coding and non-coding genes [3]. Carbohydrate antigen 19-9 (CA199) and carcinoembryonic antigen (CEA) are common biomarkers for GC diagnosis, but their sensitivity and specificity are relatively low [4]. Therefore, there is an urgent need to identify novel biomarkers for GC diagnosis.

Long non-coding RNAs (lncRNAs) are non-coding transcripts that transcribed by RNA polymerase with no protein coding capacity [5, 6]. LncRNAs participate in diverse biological processes including chromatin remodeling, transcriptional and post-transcriptional regulation [7, 8]. Increasing evidence suggest that lncRNAs play important roles in the pathogenesis of many diseases including cancer [9]. The deregulated expression of lncRNA could affect cancer cell proliferation, apoptosis, invasion and metastasis [6]. For example, lncRNA lnc-GNAT1-1 is lowly expressed in colorectal cancer and acts as a tumor suppressor through regulating RKIP-NF-κB-Snail circuit [10]. LncRNA SBF2-AS1 is highly expressed in non-small cell lung cancer and is associated with advanced tumor progression and poor prognosis in patients with non-small cell lung cancer [11, 12].

LncRNAs have been shown to be involved in GC tumorigenesis and related with the prognosis of GC patients [13]. For example, lncRNA LINC00673 is significantly upregulated in GC, and SP1-activated LINC00673 exerted oncogenic properties by functioning as a scaffold for LSD1 and EZH2 and repressing KLF2 and LATS2 expression in GC [14]. Linc00152 is highly expressed in the GC tissues and promotes GC cell proliferation by binding to EZH2 and repressing p15 and p21 expression [15–17]. Moreover, lncRNA FEZF1-AS1 is overexpressed in GC and promotes GC cell proliferation through LSD1-mediated H3K4me2 demethylation [18]. Although the previous studies have identified several lncRNAs in GC, the other lncRNAs that are involved in GC development and progression needs further investigation.

DANCR is a lncRNA required for the inhibition of the differentiation of epidermal cells [19] and the odontoblast-like differentiation of human dental pulp cells [20]. DANCR is suggested as a critical regulator of the stemness features of hepatocellular carcinoma cells [21]. In addition, DANCR is reported to promote the invasion of prostate cancer cells [22]. The increased expression of DANCR is found to be associated with advanced tumor progression and poor prognosis in colon cancer patients [23]. However, the role of DANCR in GC has not been well characterized.

In this study, we investigated the expression of DANCR in the tumor tissues and serum of GC patients, as well as the relationship between DANCR expression levels and the clinicopathological properties of GC. Moreover, the roles of DANCR in GC cell proliferation, migration, and invasion were analyzed.

## RESULTS

### DANCR is highly expressed in the tumor tissues and serum of gastric cancer patients

**Figure 1:**
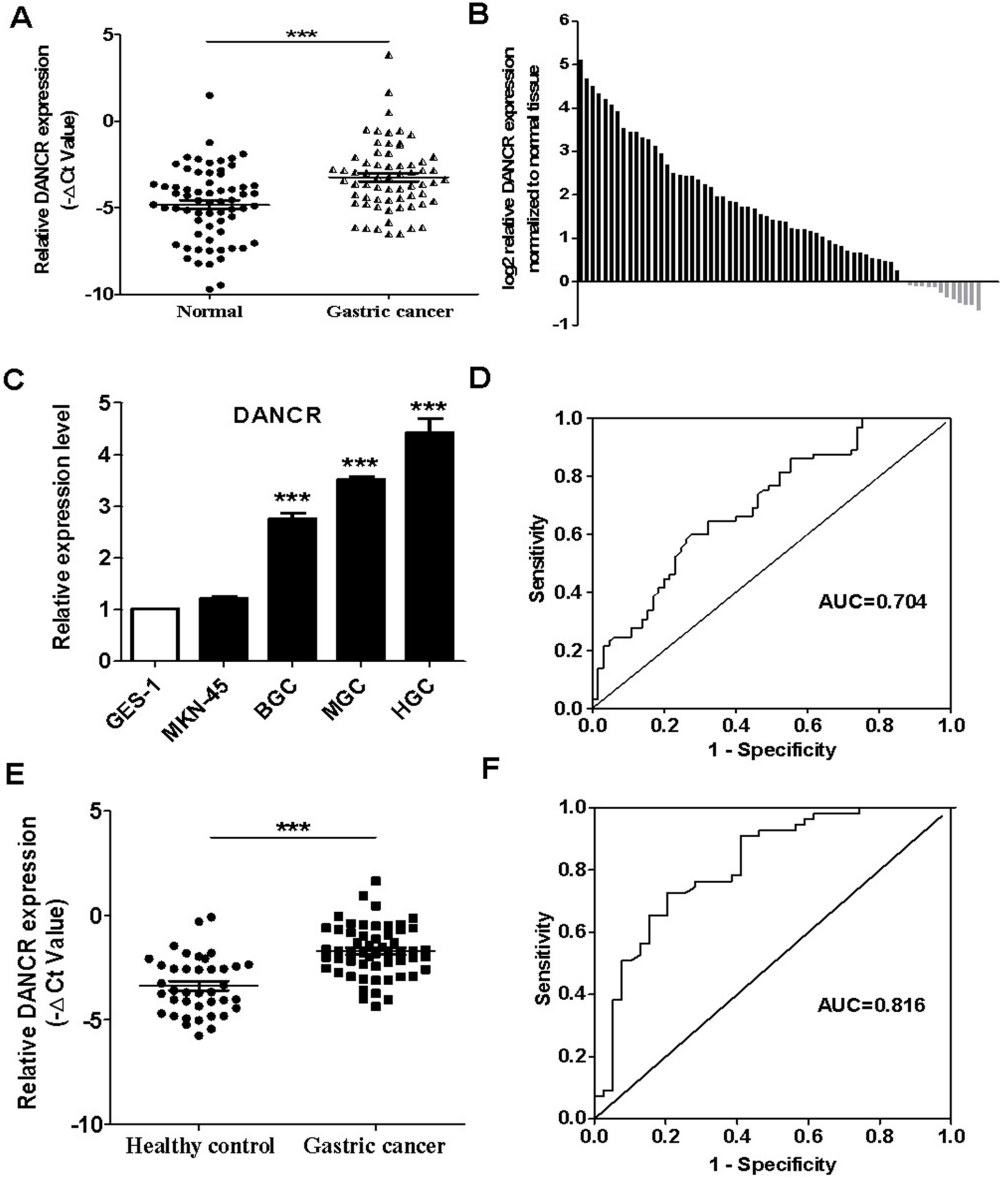
The relative expression levels of DANCR in the tumor tissues and serum samples of gastric cancer patients and gastric cancer cell lines (**A**) The relative expression levels of DANCR in gastric cancer (GC) tissues and matched adjacent normal tissues. (**B**) qRT-PCR analyses of DANCR expression in 65 paired GC tissues and adjacent normal tissues. The results were normalized to adjacent normal tissues and shown as log_2_ (2^-ΔΔCt^). (**C**) qRT-PCR analyses of DANCR expression in GC cell lines and normal gastric mucosa epithelial cells. (**D**) ROC curve for the diagnostic value of DANCR in the tumor tissues of gastric cancer patients. (**E**) qRT-PCR analyses of DANCR expression in the serum samples of gastric cancer patients (*n* = 55) and healthy controls (*n* = 39). (**F**) ROC curve for the diagnostic value of DANCR in the serum samples of gastric cancer patients. ^***^P **<** 0.001.

We first evaluated the expression of DANCR in paired GC tissues and adjacent normal tissues obtained from 65 GC patients by using qRT-PCR. As shown in Figure 1A, the expression level of DANCR was significantly higher in tumor tissues than that in adjacent normal tissues (*P* < 0.001) and 80.0% (52/65) GC tissues showed increased expression of DANCR compared to the adjacent normal tissues (Figure 1B). We also examined DANCR expression in normal gastric mucosa epithelial cell line (GES-1) and GC cell lines (BGC-823, MGC-803, HGC-27 and MKN-45). DANCR expression was also upregulated in most GC cell lines compared to that in normal gastric mucosa epithelial cell line (Figure 1C). We then analyzed the relationship between DANCR expression and the clinicopathological features. The GC patients with high expression levels of DANCR were prone to have large tumor (*P* = 0.001), lymph node metastasis (*P* = 0.000), invasion depth (*P* = 0.028) and advanced TNM stage (*P* = 0.009) (Table 1). We developed a receiver operating curve (ROC) to investigate the diagnostic value of DANCR in GC tissues. The area under the ROC curve (AUC) was 0.704 (95% confidence interval (CI), 0.616-0.793, *P* < 0.001, Figure 1D) and the sensitivity and specificity were 64.6% and 67.7%, respectively.

**Table 1:** The association between DANCR expression levels (–ΔCt) in tumor tissues and the clinicopathological features of gastric cancer patients

Features	Number	DANCR expression		Mean ± SD	*P* value
		High	Low		
**Gender**					
Male	47	28	19	−3.13 ± 2.08	0.599
Female	18	12	6	−3.52 ± 1.75	
**Age (years)**					
**<** 60	19	10	9	−3.25 ± 2.44	0.343
≥60	46	30	16	−3.24 ± 1.80	
**Tumor size(cm)**					
**<** 5	32	13	19	−3.17 ± 2.04	0.001
≥5	33	27	6	−3.31 ± 1.97	
**Differentiation1**					
Moderate	20	13	7	−2.70 ± 2.60	0.679
Poor	42	25	17	−3.48 ± 1.68	
**Lymphatic metastasis**					
N0	17	4	13	−3.87 ± 1.38	0.000
N1−3	48	36	12	−3.02 ± 2.13	
**Distal metastasis**					
M0	64	39	25	−3.19 ± 1.97	1.000
M1	1	1	0	−6.14	
**Venous or Perineural invasion**					
Absent	43	26	17	−3.34 ± 1.80	0.804
Present	22	14	8	−3.05 ± 2.35	
**Invasion depth**					
T1 and T2	6	1	5	−4.45 ± 1.16	0.028
T3 and T4	59	39	20	−3.12 ± 2.02	
**TNM stage**					
I and II	19	7	12	−3.74 ± 1.35	0.009
III and IV	46	33	13	−3.03 ± 2.18	
**Tumor location**					
Antrum	16	11	5	−2.54 ± 2.67	0.385
Body	6	4	2	−2.92 ± 1.83	
Angulus	6	3	3	−3.05 ± 1.50	
Cardia	18	8	10	−3.72 ± 1.77	
Others	19	14	5	−3.53 ± 1.68	

TNM tumor−node−metastasis. 1 indicates missing 3 cases.

To further explore the potential utility of DANCR for GC diagnosis, we detected the expression of DANCR in serum samples of GC patients (*n* = 55) and healthy controls (*n* = 39). The results of qRT-PCR showed that the expression of DANCR in the serum of GC patients was higher than that in the serum of healthy controls (*P* < 0.001, Figure 1E). The correlation between serum DANCR expression levels and the clinicpathological characteristics was analyzed and presented in Table 2. We found that the serum levels of DANCR were associated with tumor size (*P* = 0.000), lymphatic metastasis (*P* = 0.000), invasion depth (*P* = 0.017) and TNM stage (*P* = 0.000). To further understand the diagnostic value of serum DANCR in GC, ROC curve was constructed. The AUC of serum DANCR was 0.816 (95% CI, 0.727-0.905, *P* < 0.001). The sensitivity of serum DANCR expression was 72.7%, with the specificity of 79.5% (Figure 1F). Moreover, the expression level of serum DANCR in GC is relatively stable (data not shown). Taken together, these data indicate that the expression level of DANCR is elevated in the tumor tissues and serum of gastric cancer patients and the increased expression of DANCR is associated with the malignant progression of gastric cancer.

**Table 2:** The correlation between serum DANCR expression levels (–ΔCt) and the clinicopathological characteristics of gastric cancer patients

Features	Number	DANCR expression		Mean ± SD	*P* value
		High	Low		
**Gender**					
Male	44	33	11	−1.74 ± 1.17	0.468
Female	11	7	4	−1.60 ± 1.55	
**Age (years)**					
**<** 60	15	10	5	−1.63 ± 1.46	0.735
≥60	40	30	10	−1.74 ± 1.17	
**Tumor size(cm)**					
**<** 5	28	13	15	−2.20 ± 1.38	0.000
≥5	27	27	0	−1.21 ± 0.85	
**Differentiation1**					
Moderate	20	13	7	−1.71 ± 1.50	0.363
Poor	34	26	8	−1.75 ± 1.09	
**Lymphatic metastasis**					
N0	15	4	11	−2.87 ± 1.02	0.000
N1−3	40	36	4	−1.28 ± 1.03	
**Venous or Perineural invasion**					
Absent	39	28	11	−1.77 ± 1.23	1.000
Present	16	12	4	−1.58 ± 1.30	
**Invasion depth**					
T1 and T2	3	0	3	−3.69 ± 0.64	0.017
T3 and T4	52	40	12	−1.60 ± 1.17	
**TNM stage**					
I and II	16	6	10	−2.46 ± 1.30	0.000
III and IV	39	34	5	−1.41 ± 1.10	

^1^indicates missing 2 cases.

### DANCR knockdown inhibits the proliferation of GC cells *in vitro* and *in vivo*

**Figure 2:**
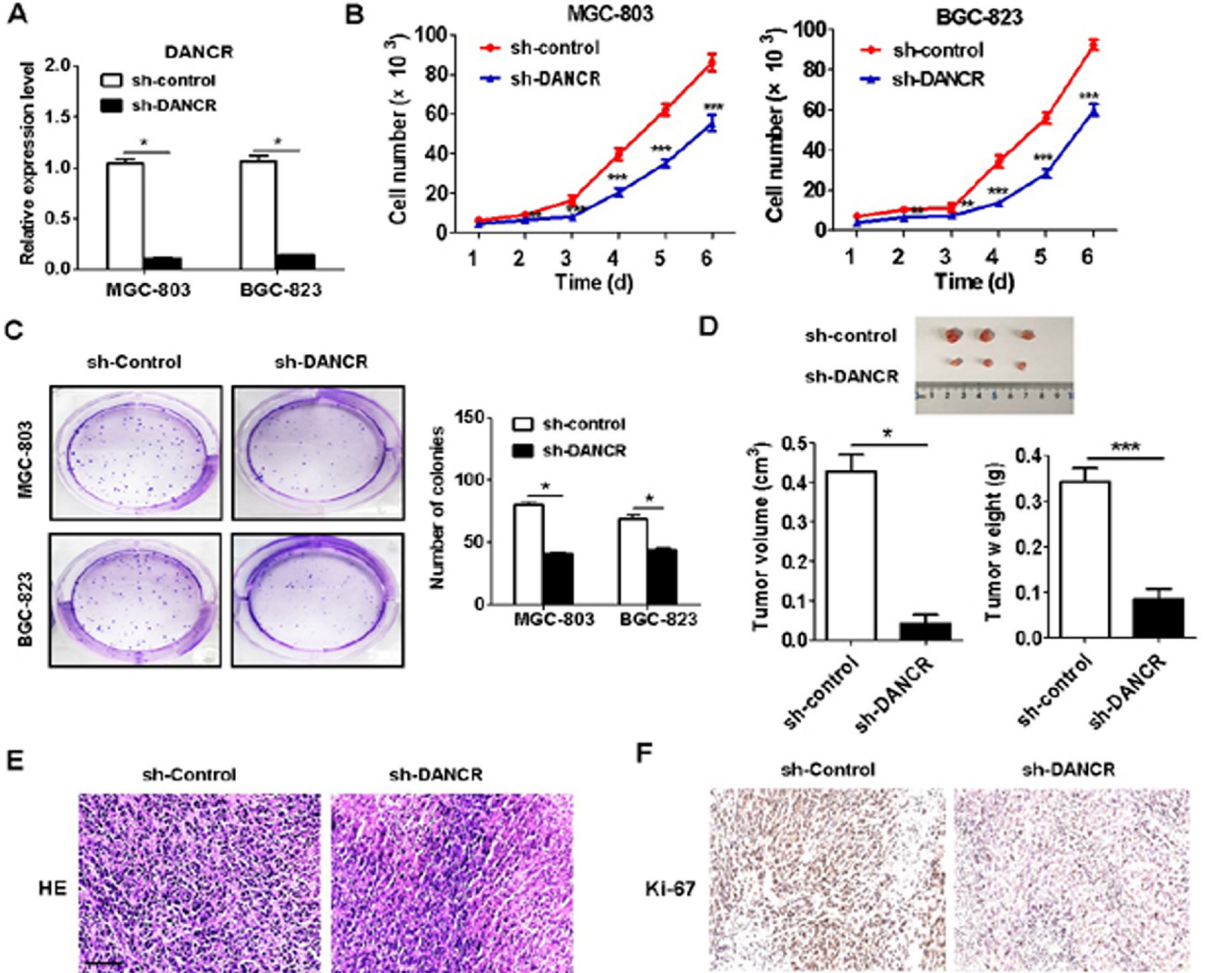
DANCR knockdown inhibits the proliferation of gastric cancer cells *in vitro* and *in vivo* (**A**) qRT-PCR analyses of DANCR expression in MGC-803 and BGC-823 cells transfected with sh-Control and sh-DANCR. (**B**) Cell counting assays for the growth of sh-DANCR transfected MGC-803 and BGC-823 cells. (**C**) Colony formation assays were used to determine the proliferation of MGC-803 and BGC-823 cells transfected with sh-DANCR. (**D**) The tumor volumes and tumor weights of mice inoculated with sh-Control and sh-DANCR transfected MGC-803 cells. (**E**) HE staining for tumor tissues of mice inoculated with sh-Control and sh-DANCR transfected MGC-803 cells. (**F**) Immunohistochemical staining of Ki-67 in the tumor tissues of mice inoculated with sh-Control and sh-DANCR transfected MGC-803 cells. ^*^P < 0.05; ^**^P < 0.01; ^***^P < 0.001.

To determine the functional roles of DANCR in gastric cancer, we knocked down DANCR expression in GC cell lines MGC-803 and BGC-823 by using shRNA. The knockdown efficiency of DANCR in the transfected cells was verified by using qRT-PCR (Figure 2A).The results of cell counting assay revealed that DANCR knockdown significantly impaired the growth GC cells (Figure 2B). In similar, the results of colony formation assay also revealed that DANCR knockdown remarkably inhibited the colony formation abilities of MGC-803 and BGC-823 cells (Figure 2C). The results of *in vivo* xenograft tumor model showed that the mice injected with sh-DANCR MGC-803 cells developed smaller sizes of tumors than that injected with sh-control MGC-803 cells (Figure 2D). The percentage of ki-67-positive cells was lower in the tumor tissues of mice in sh-DANCR group compared to that in sh-control group (Figures 2E and 2F). These findings suggest that DANCR knockdown inhibits the proliferation of GC cells *in vitro* and *in vivo*.

### DANCR knockdown induces cell cycle arrest and cell apoptosis in GC cells

**Figure 3:**
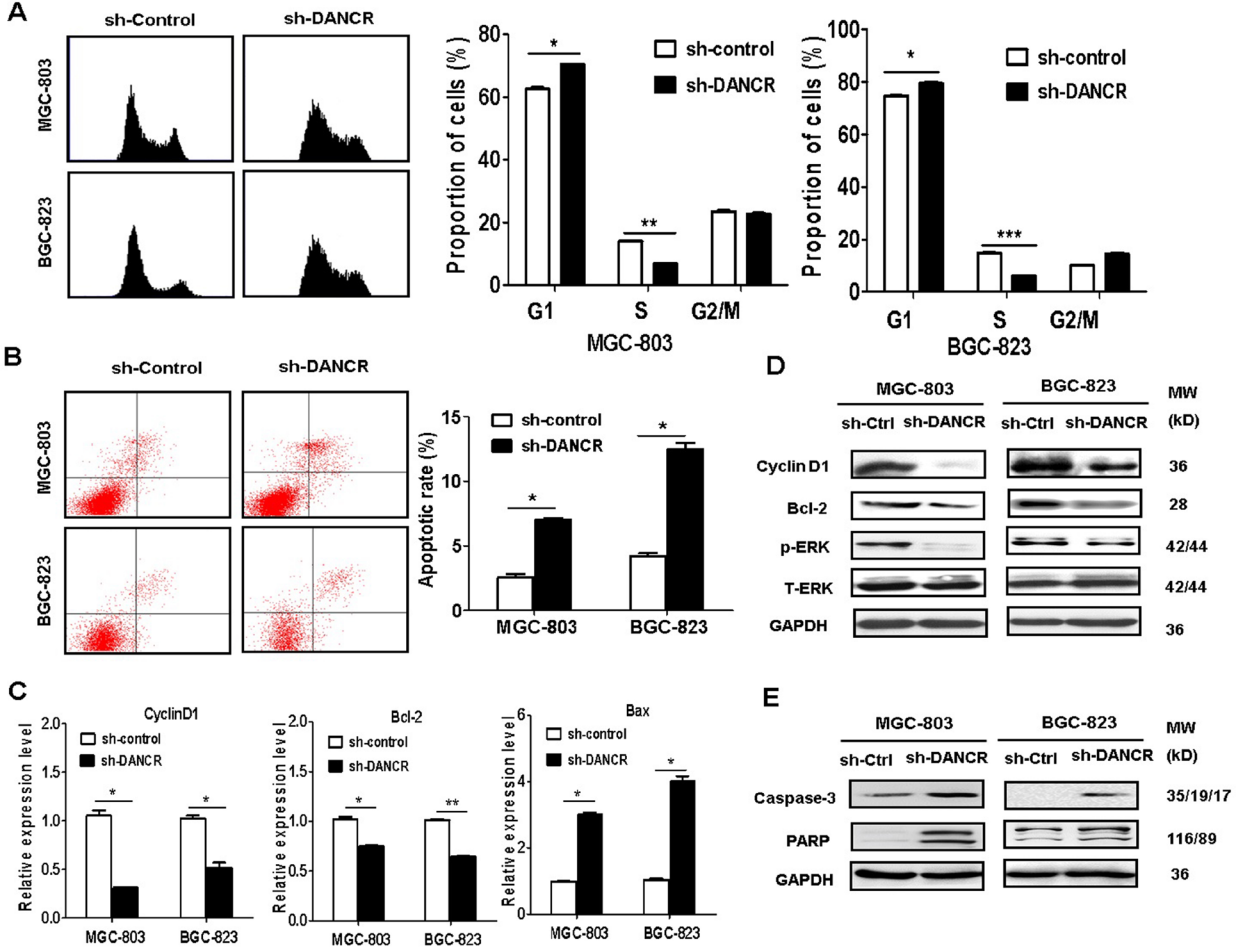
DANCR knockdown induces cell cycle arrest and cell apoptosis (**A**) Flow cytometric analyses of cell cycle distribution in sh-DANCR transfected MGC-803 and BGC-823 cells. (**B**) Flow cytometric analyses of cell apoptosis in sh-DANCR transfected MGC-803 and BGC-823 cells. (**C**) qRT-PCR analyses of the expression of cell cycle- and apoptosis-related genes in sh-DANCR transfected MGC-803 and BGC-823 cells. (**D**) Western blotting assays for the expression of cell cycle-related proteins in sh-DANCR transfected MGC-803 and BGC-823 cells. (**E**) Western blotting assays for the expression of apoptosis-related proteins in sh-DANCR transfected MGC-803 and BGC-823 cells. ^*^P < 0.05, ^**^P < 0.01, ^***^P < 0.001.

We then examined the impact of DANCR knockdown on cell cycle in GC cells. The results of flow cytometric analyses showed a decrease in the percentage of cells at S phase and an accumulation in the percentage of cells at G1 phase in DANCR knockdown groups in comparison with control groups (Figure 3A). We also determined the effects of DANCR knockdown on cell apoptosis in GC cells. As shown in Figure 3B, DANCR knockdown led to an increase in the percentage of apoptotic cells in both MGC-803 and BGC-823 cells. The results of quantitative RT-PCR analyses showed that the expression of proliferation-related genes including cyclin D1 and Bcl2 was downregulated while that of apoptosis-related gene Bax was upregulated in DANCR knockdown gastric cancer cells (Figure 3C). The results of western blot analyses showed that the expression of cyclin D1 and Bcl2 was downregulated while that of the active forms of caspase-3 ad PARP was upregulated in DANCR knockdown gastric cancer cells (Figures 3D–3E). Altogether, these results suggest that DANCR knockdown inhibits the proliferation of GC cells by inducing cell cycle arrest and cell apoptosis.

### DANCR knockdown inhibits the migration and invasion of GC cells

**Figure 4:**
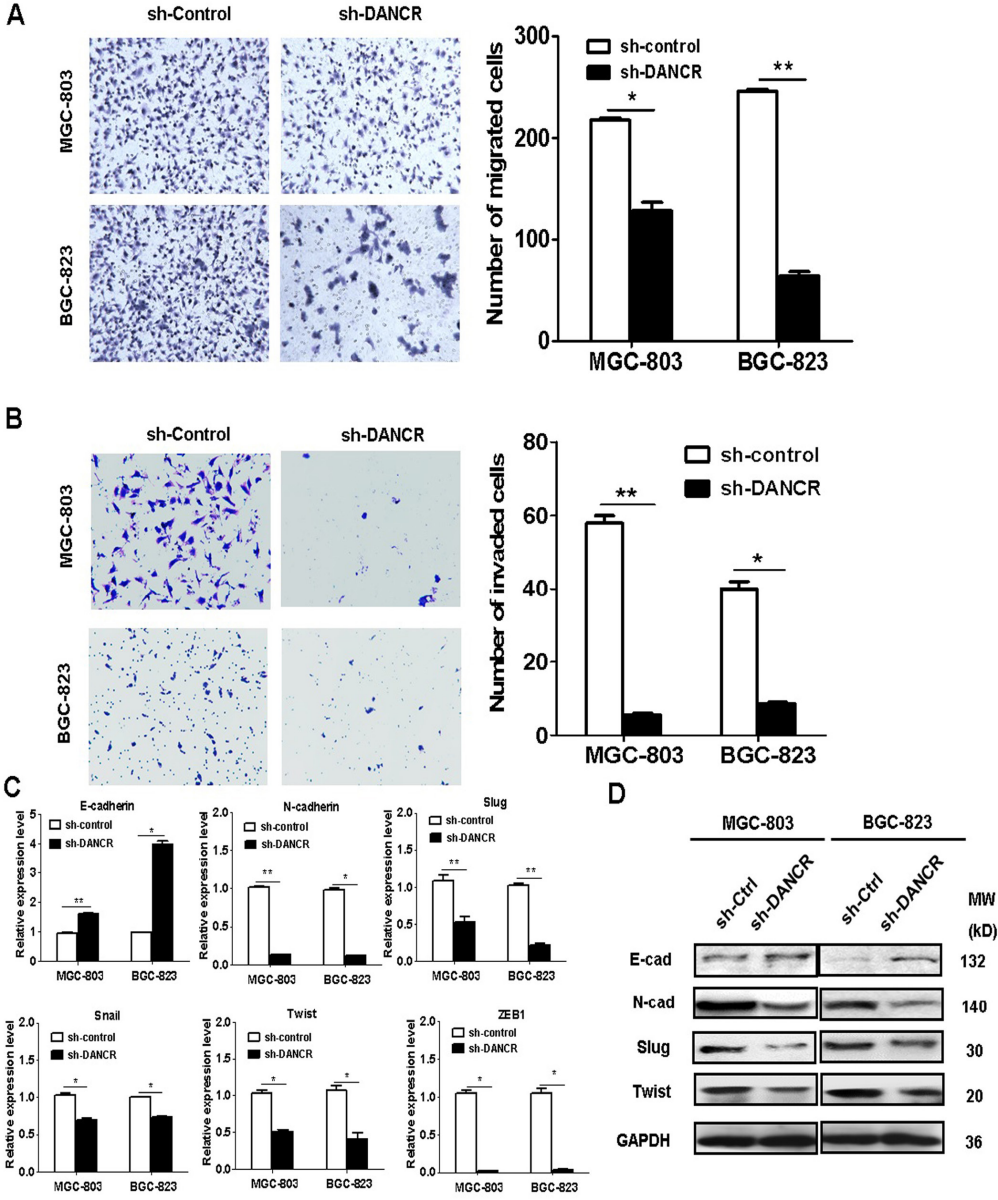
DANCR knockdown inhibits the migration and invasion of gastric cancer cells (**A**) Transwell migration assays were performed to determine the migratory abilities of sh-DANCR transfected MGC-803 and BGC-823 cells. (**B**) Matrigel invasion assays were performed to determine the invasive abilities of sh-DANCR transfected MGC-803 and BGC-823 cells. (**C**) qRT-PCR analyses of the expression of EMT-related genes in sh-DANCR transfected MGC-803 and BGC-823 cells. (**D**) Western blotting assays for the expression of EMT-related proteins in sh-DANCR transfected MGC-803 and BGC-823 cells. ^*^P < 0.05, ^**^P < 0.01.

We next wanted to test the effects of DANCR on the migratory and invasive abilities of GC cells. The results of transwell migration assays showed that the number of migrated cells was lower in sh-DANCR group than that in sh-control group (Figure 4A). In similar, the results of matrigel invasion assays showed that the number of invaded cells decreased in sh-DANCR group compared to that in sh-control group (Figure 4B). We then tested the effects of DANCR knockdown on the process of epithelial-mesenchymal transition (EMT), a critical step for cancer metastasis. We measured the mRNA and protein levels of EMT related markers in DANCR knockdown GC cells. As shown in Figure 4C, the knockdown of DANCR in GC cells increased the mRNA expression of E-cadherin while decreased that of N-cadherin, slug, snail, twist and ZEB-1. In addition, the results of western blot analyses showed that DANCR knockdown inhibited the protein level of E-cadherin while decreased that of N-cadherin, slug, and twist. These results suggest that DANCR may promote the migration and invasion of gastric cancer cells through the induction of EMT.

### DANCR overexpression promotes the proliferation, migration, and invasion of GC cells

**Figure 5:**
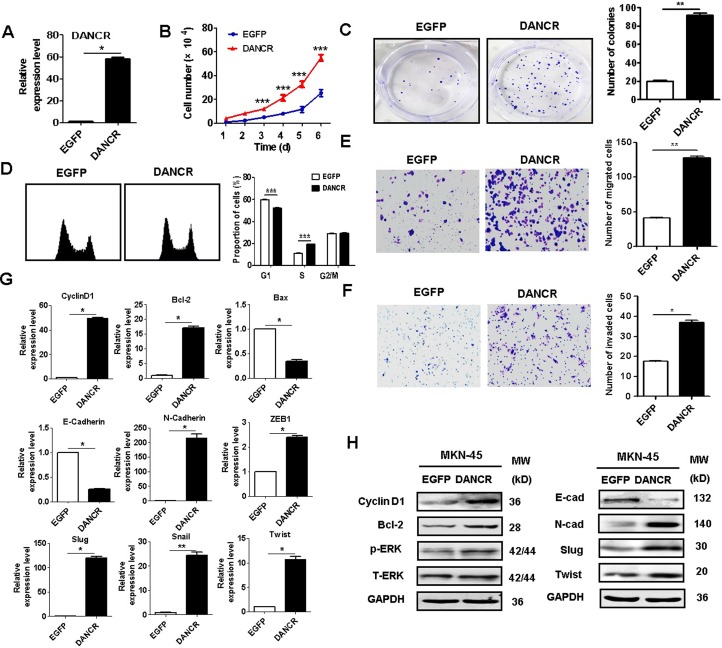
DANCR overexpression promotes the proliferation, migration, and invasion of gastric cancer cells (**A**) qRT-PCR analyses of DANCR expression in MKN45 cells transfected with DANCR. (**B**) Cell counting assays for the growth of DANCR-overexpressing MKN-45 cells. (**C**) Colony formation assays for the proliferation of DANCR-overexpressing MKN-45 cells. (**D**) Flow cytometric analyses of cell cycle distribution in DANCR-overexpressing MKN-45 cells. (**E**) Transwell migration assays were performed to determine the migratory abilities of DANCR-overexpressing MKN-45 cells. (**F**) Matrigel invasion assays were performed to determine the invasive abilities of DANCR-overexpressing MKN-45 cells. (**G**) qRT-PCR analyses of the expression of cell cycle-, apoptosis-, and EMT-related genes in DANCR-overexpressing MKN-45 cells. (**H**) Western blotting assays for the expression of cell cycle-, apoptosis-, and EMT-related proteins in DANCR-overexpressing MKN-45 cells. ^*^P < 0.05, ^**^P < 0.01, ^***^P < 0.001.

To further demonstrate the oncogenic roles of DANCR in gastric cancer, we overexpressed DANCR in MKN45 cells that harbor relatively low level of DANCR. The efficiency of DANCR overexpression was confirmed by using qRT-PCR (Figure 5A). The results of cell counting assay and cell colony formation assay showed that DANCR overexpression obviously promoted the proliferation of MKN45 cells (Figure 5B–5C). DANCR overexpression induced an increase in the percentage of cells at S phase in MKN45 cells (Figure 5D). In addition, DANCR overexpression enhanced the migratory and invasive abilities of MKN45 cells (Figure 5E–5F). As shown in Figure 5G, the expression of cyclin D1, Bcl2, N-cadherin, slug, twist, and ZEB1 genes was increased while that of E-cadherin and Bax was decreased in DANCR-overexpressing MKN45 cells. DANCR overexpression also upregulated the protein levels of cyclin D1, Bcl2, N-cadherin, slug, and twist while downregulated that of E-cadherin in MKN-45 cells (Figure 5H). In summary, these results suggest that DANCR overexpression promotes the proliferation, migration and invasion of gastric cancer cells.

### DANCR is activated by SALL4 in GC cells

**Figure 6:**
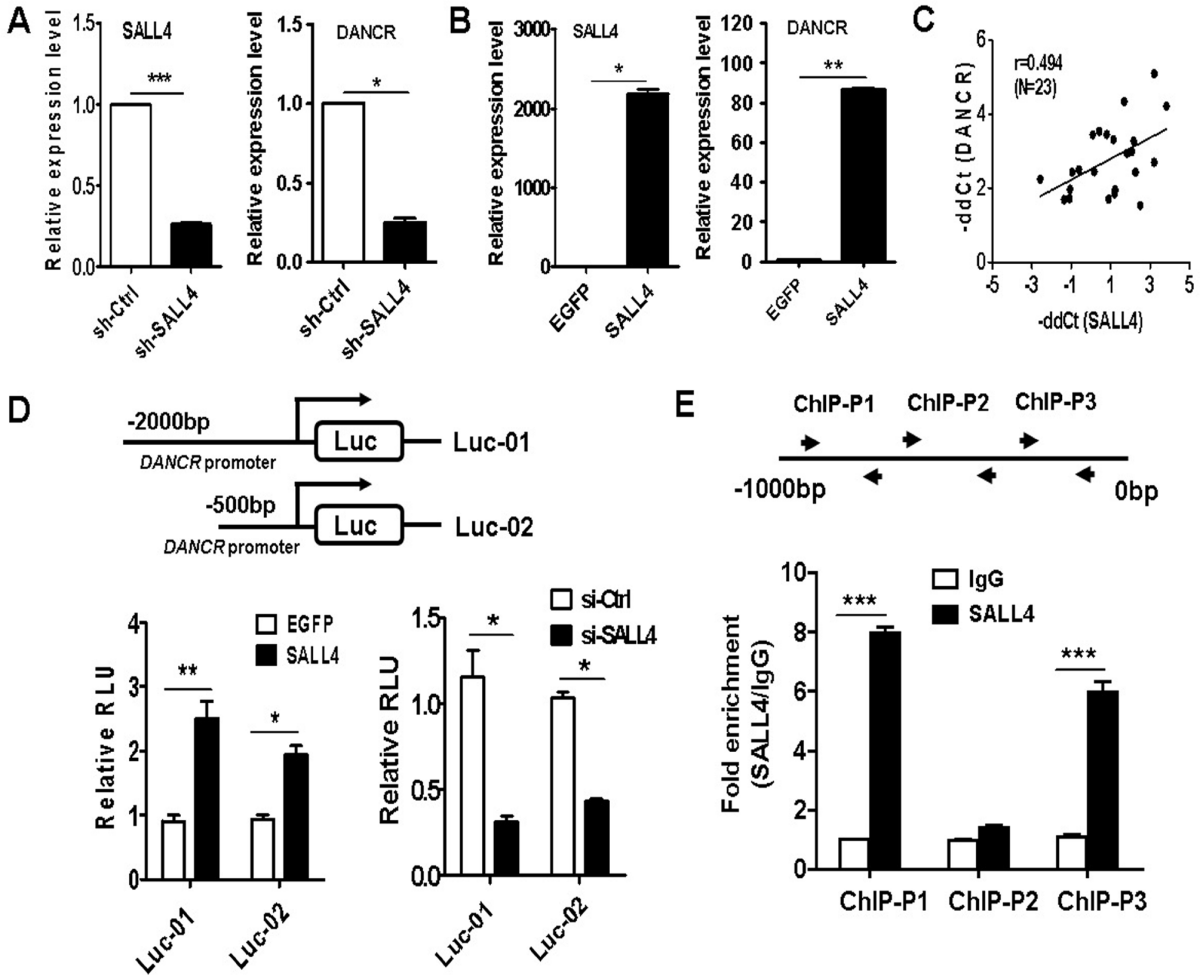
DANCR is activated by SALL4 in gastric cancer cells (**A**) qRT-PCR of DANCR expression in sh-SALL4 transfected MGC-803 cells. (**B**) qRT-PCR of DANCR expression in SALL4-overexpressing MKN-45 cells. (**C**) The expression of SALL4 and DANCR in gastric cancer tissues was determined by using qRT-PCR. The correlation between SALL4 and DANCR expression levels was determine by Pearson correlation analysis. (**D**) MGC-803 cells were co-transfected with DANCR luciferase reporter vector and SALL4-overexpressing plasmid or SALL4 knockdown siRNA. The luciferase activity was evaluated by using dual-luciferase reporter system. (**E**) ChIP-PCR analyses of SALL4 binding to DANCR promoter in MGC80-3 cells. ^*^P < 0.05, ^**^P < 0.01, ^***^P < 0.001.

DANCR functions as a regulator of cell stemness in human hepatocellular carcinoma [21]. We have previously shown that SALL4 (sal-like protein 4) is a critical transcription factor that regulates the stemness of gastric cancer cells [24]. Thus, we wanted to know whether SALL4 regulates DANCR expression in GC. We first determined the association between the expression of SALL4 and DANCR in GC cells. As shown in Figure 6A–6B, SALL4 knockdown decreased while SALL4 overexpression increased the expression levels of DANCR. In addition, we showed that the expression of SALL4was positively associated with that of DANCR in the tumor tissues of GC patients (Figure 6C). To demonstrate that SALL4 directly regulates DANCR expression, we cloned the promoter of DANCR gene and inserted it into a luciferase reporter vector. The luciferase reporter vector was co-transfected with SALL4-overexressing plasmid or SALL4 siRNA into MGC-803 cells. The results of luciferase reporter assays showed that SALL4 overexpression upregulated while SALL4 knockdown downregulated the activity of DANCR luciferase reporter vector (Figure 6D). We then performed a chromatin immunoprecipitation assay to determine the binding of SALL4 protein to the promoter region of DANCR gene. The chromatin was immunoprecipitated by IgG or SALL4-specific antibodies for PCR detection. The results of ChIP assay showed an obvious enrichment of the chromatin in SALL4 group but not in IgG group (Figure 6E), suggesting that SALL4 may regulate DANCR expression by binding to its promoter.

### DANCR exerts its oncogenic activities in GC cells through the activation of β-catenin pathway

**Figure 7:**
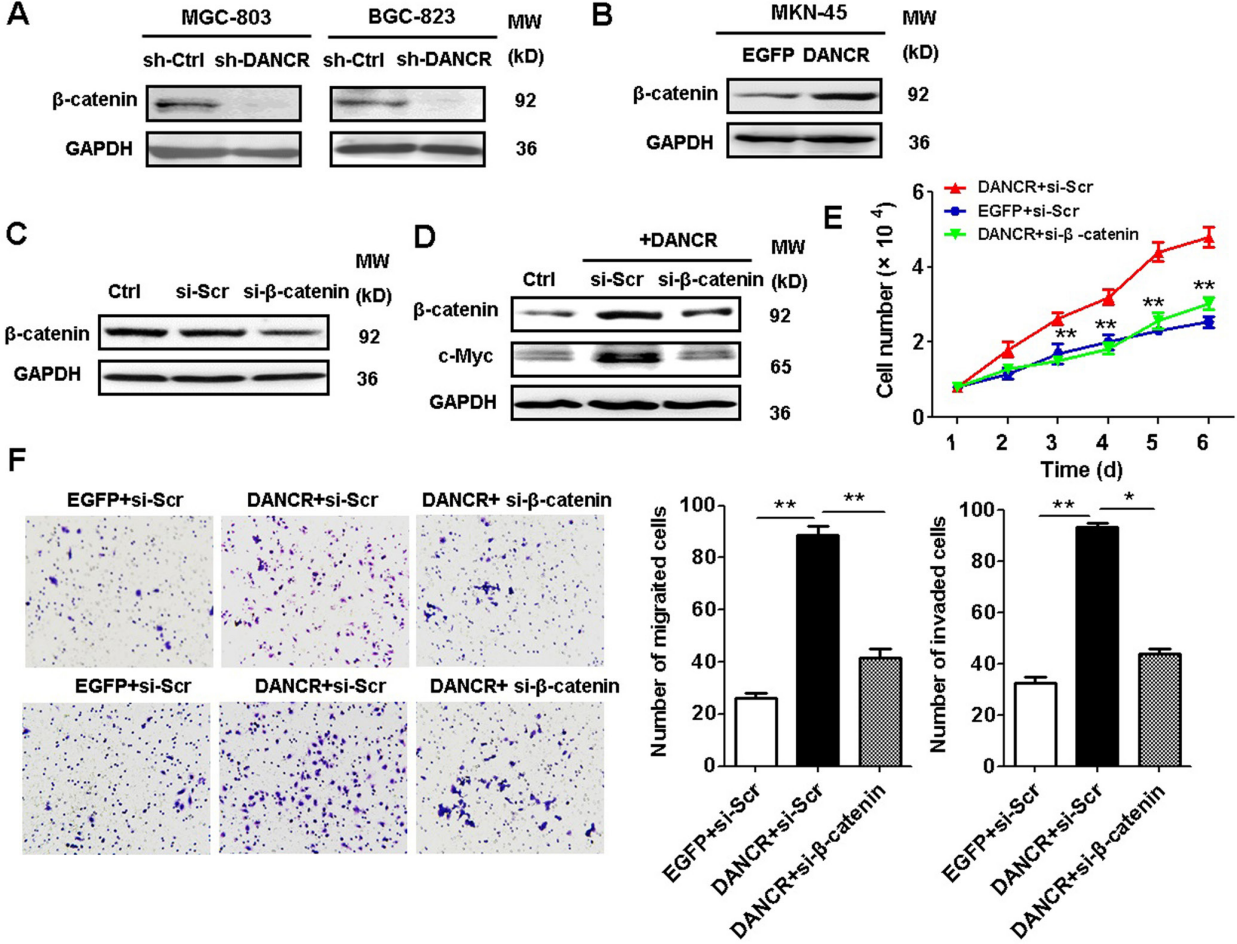
DANCR activates β-catenin pathway to promote gastric cancer progression (**A**) Western blotting assays for the expression of β-catenin in sh-DANCR transfected MGC-803 and BGC-823 cells. (**B**) Western blotting assays for the expression of β-catenin in DANCR-overexpressing MKN-45 cells. (**C**) Western blotting analyses of β-catenin expression in MKN-45 cells transfected with β-catenin siRNA. (**D**) Western blotting analyses of β-catenin and c-Myc expression in MKN-45 cells co-transfected with DANCR-overexpressing plasmid and β-catenin siRNA. (**E**) Cell counting assays for the growth of MKN-45 cells co-transfected with DANCR-overexpressing plasmid and β-catenin siRNA. (**F**) Transwell migration assays and matrigel invasion assays for the migration and invasion abilities of MKN-45 cells co-transfected with DANCR-overexpressing plasmid and β-catenin siRNA. ^*^P < 0.05, ^**^P < 0.01.

DANCR acts a regulator of β-catenin in hepatocellular carcinoma cells [25]. We then determined the expression of β-catenin in DANCR knockdown and DANCR overexpressing GC cells. We found that DANCR knockdown decreased while DANCR overexpression increased the expression of β-catenin in GC cells (Figure 7A–7B). To confirm the importance of DANCR-mediated upregulation of β-catenin, we knocked down β-catenin in DANCR-overexpressing GC cells. As shown in Figure 7C–7D, the increased expression of β-catenin and its target gene c-Myc by DANCR was inhibited by β-catenin siRNA in GC cells. In addition, the promoting role of DANCR in GC cell proliferation, migration and invasion were also decreased by β-catenin knockdown in GC cells (Figure 7E–7F), suggesting that DANCR activates β-catenin to promote gastric cancer progression.

## DISCUSSION

LncRNAs play active roles in tumor initiation, development and progression. The aberrant expression of lncRNAs is observed in many human cancers, including colorectal cancer, liver cancer, and gastric cancer [26–28]. DANCR is initially reported to regulate the differentiation of progenitor cells [19]. The recent studies suggest that DANCR expression is upregulated in hepatocellular carcinoma [21], prostate cancer [22], and colorectal cancer [23]. In this study, we reported that DANCR was also upregulated in GC tissues and this result was consistent with that reported by Hao *et al.* [29]. We found that the upregulation of DANCR was positively correlated with tumor size, lymphatic metastasis, invasion depth and advanced TNM stage, indicating that DANCR overexpression may contribute to the development and progression of gastric cancer.

The deregulated expression of lncRNAs could be detected in the body fluids including plasma, serum and urine [30]. The circulating lncRNAs have been suggested as ideal biomarkers for the diagnosis and prognosis prediction of gastric cancer [31, 32]. Our study is the first to quantify DANCR in the serum of GC patients. We found that the expression level of DANCR was also elevated in the serum of GC patients compared to that in healthy controls. The expression levels of DANCR were significantly correlated with tumor size, lymphatic metastasis, invasion depth and TNM stage, suggesting that serum DANCR may be used as an easier and convenient diagnosis biomarker for GC. The previous studies have shown that DANCR could be detected in the extracellular vesicles (EV) from human breastmilk [33] and in the circulating monocytes [34]. Whether DANCR is stored in EV form or in a free form in the serum deserves further investigation. In addition, the increased DANCR expression in the serum originates from tumor expansion (tumor cells derived) or excessive inflammatory response (monocytes derived) is another important question to be addressed. Further studies should focus on large-scale sample analysis and long-term follow-up to explore the potential value of DANCR in GC.

DANCR maintains the stemness features of hepatocellular carcinoma cells [21] and promotes the invasion of prostate cancer cells [22]. Gain- and loss-of-function studies showed that DANCR knockdown inhibited while DANCR overexpression promoted GC cell proliferation, migration, and invasion. In vivo animal studies conformed that DANCR knockdown retarded tumor growth. DANCR depletion inhibited GC cell proliferation by inducing cell cycle arrest and cell apoptosis. EMT is an important process of tumor metastasis and involves a set of transcription factors [35–37]. Cells undergoing EMT express low level of epithelial markers and high level of mesenchymal markers [38]. We observed that DANCR knockdown downregulated the expression of EMT regulators such as slug and twist and increased the expression of E-cadherin in GC cells while DANCR overexpression had the opposite effect. Our findings, together with those from the others, suggest that DANCR may promote gastric cancer progression via regulating cell proliferation, migration and invasion.

The upregulation of lncRNAs in cancer is associated with increased activities of oncogenic transcription factors such as c-Myc, SP1 and E2F1. Kim *et al.* suggest that MYCLo-2, a myc-regulated lncRNA, promotes the proliferation of colorectal cancer cells by inhibiting the expression of p21 and p15 [39]. Huang *et al.* demonstrate that LINC00673 is activated by SP1 and promotes the proliferation and invasion of gastric cancer cells [14]. DANCR has been reported to be upregulated by Sox4 to promote the proliferation of synovium-derived mesenchymal stem cells (SMSCs) [40]. In this study, we identified DANCR as a target of SALL4 protein in GC cells. SALL4 is a zinc finger transcription factor that governs the self-renewal and pluripotency of embryonic stem cells. The recent studies suggest that SALL4 plays active roles in tumor growth, metastasis and therapy resistance [41]. We have previously shown that SALL4 is critically involved in gastric cancer progression by regulating cell stemness and EMT [24, 42]. The regulation of DANCR may contribute, at least in part, to the oncogenic roles of SALL4 in GC.

In human hepatocellular carcinoma cells, DANCR could bind to β-catenin mRNA and enhance its expression [21]. In human prostate cancer cells, DANCR binds to EZH2 and inhibits the expression of TIMP-2 by epigenetic silencing [22]. In human osteosarcoma cells, DANCR could upregulate AXL expression via miR-33a-5p inhibition [43]. The abnormal activation of β-catenin frequently occurs in human cancers and has been associated with the promotion of tumor growth and metastasis [44, 45]. We reported in this study that DANCR upregulated the expression of β-catenin and activated its downstream target genes in GC cells, promoting gastric cancer cell proliferation, migration and invasion. Whether other mechanisms are responsible for the functional roles of DANCR in GC needs further investigation.

In conclusion, we demonstrate in this study that DANCR is upregulated in the tumor tissues and serum of GC patients. The elevated expression of DANCR is associated with malignant progression of gastric cancer. DANCR promotes gastric cancer cell proliferation, migration and invasion by accelerating cell cycle progression, inhibiting apoptosis, and inducing EMT. DANCR is upregulated by SALL4 and exerts its tumor-promoting roles partly through the activation of β-catenin pathway. Our findings indicate that DANCR plays an oncogenic role in gastric cancer and have the potential to serve as a novel diagnostic biomarker.

## MATERIALS AND METHODS

### Clinical samples

Sixty-five pairs of GC tissues and adjacent normal tissues were obtained from patients who had underwent surgery in the Department of General Surgery, the Affiliated People's Hospital of Jiangsu University between April 2015 and June 2016 for this study. The adjacent normal tissues were obtained from tissues that were located 5 cm away from the edge of GC. Peripheral whole-blood samples from GC patients (*n* = 55) and healthy controls (*n* = 39) were collected in EDTA anticoagulation tubes and centrifuged at 3000 *g* for 10 min. The sera were carefully transferred to RNase-free tubes. All specimens were immediately frozen in liquid nitrogen and stored at –80°C before use. None of these patients had received any treatment before surgery. GC diagnosis was histopathologically confirmed and tumor stage was evaluated according to tumor, node and metastasis (TNM) classification system of the International Union Against Cancer (7th, ed. 2010). This study was approved by the Institutional Ethical Committee of Jiangsu University, and signed informed consent was obtained from all patients.

### Cell culture

Gastric cancer cell lines (BGC-823, MGC-803, HGC-27 and MKN-45) were purchased from the Cell Bank of the Chinese Academy of Sciences (Shanghai, China). The normal gastric mucosa epithelial cell line GES-1 was obtained from Gefan Biological Technology (Shanghai, China). MGC-B03 cells were cultured with high glucose-DMEM containing 10% fetal bovine serum (FBS; Gibco). GES-1, BGC-823, MKN-45, and HGC-27 cells were cultured with RPMI 1640 medium (Invitrogen) supplemented with 10% FBS. All the cells were cultured at 37 °C in a 5% CO2 humidified incubator.

### RNA interference and gene overexpression

The shRNA against DANCR was purchased from Hanbio (Shanghai, China). The sequences of sh-DANCR and the shRNA control (sh-Ctrl) were shown in Supplementary Table 1. The siRNAs against SALL4 and β-catenin were produced by Genepharrm (Suzhou, Jiangsu, China). The sequences of siRNAs were shown in Supplementary Table 2. MGC-803, BGC-823, and MKN-45 cells (1×10^5^ cells/well) were grown in 6-well plates and transfected by using HB-TRLF-1000 LipoFiter transfection reagent (Hanbio) for 36 hours.

### RNA extraction and quantitative real-time polymerase chain reaction (qRT-PCR)

Total RNA was extracted from frozen tumor/normal tissues using Ultrapure RNA Kit following the manufacturer's instructions (Cwbio, Beijing, China). Total RNA in the freshly cultured cells was extracted by using Trizol (Gibco).Total RNA in serum was purified by using miRNeasy serum/plasma kit according to the manufacturer's procedures (Qiagen). The concentration and purity of total RNA was evaluated by using NanoDrop 2000 spectrophotometer (Thermo). RNA was reversely transcribed into cDNA by using the HiScript 1st strand cDNA synthesis kit (Vazyme) following the manufacturer's instructions. QRT-PCR was performed with UltraSYBR Mixture (Cwbio) on a CFX96 Real-time PCR Detection System (Bio-Rad). Small nucleolar RNA U6 was used as reference for DANCR to obtain the relative threshold cycle (Ct) values, and then calculated by using the 2^-△△Ct^ method. The sequences of the specific primers were shown in Supplementary Table 3.

### Cell counting and colony formation assay

The transfected cells (1000 cells/well) were seeded into 24-well plates and the cells were collected and counted for consecutive 6 days. For the colony formation assay, the transfected cells were placed in 6-well plates (1000 cells/well) and incubated at 37°C in a 5% CO2 humidified incubator. The culture medium was changed every 3 days. After 10 days, the cells were fixed with 4% paraformaldehyde and stained with crystal violet. Visible colonies were counted and photographed. All the results are the mean values of three independent experiments.

### Transwell migration assay

The transfected cells in serum-free medium were placed into the upper transwell chambers with inserts of 8 μm pore size. The medium with 10% FBS was added to the bottom chamber. After incubation for 16 hours, the cells that migrated through the membrane to the lower surface were fixed with paraformaldehyde, stained with crystal violet, counted and photographed. Experiments were carried out in triplicate independently.

### Cell cycle analysis

The transfected cells were harvested and washed with PBS, stained with 100 μg/ml propidium iodide (PI) at room temperature for 30 min, and analyzed by using flow cytometry. The percentage of the cells in different phases was counted and compared.

### Cell apoptosis assay

The transfected cells were collected and washed with PBS. The cells were stained with Annexin V-APC/PI at room temperature for 15 min. The percentage of apoptotic cells were analyzed by using flow cytometry.

### *In vivo* animal study

The procedures for animal studies were approved by the Animal Use Committee of Jiangsu University. Male athymic Balb/c nude mice (aged four weeks) were purchased from Shanghai Animal Center (Shanghai, China). The mice were housed in a pathogen-free animal facility. The mice were randomly assigned to control and DANCR knockdown groups (five mouse per group) and injected subcutaneously with MGC-803 cells (2 × 10^6^ cells/mouse; in 0.2 ml PBS). Tumor volume was determined every three days by the formula: 0.5 × length × width^2^.

### Immunohistochemistry

All the mice were sacrificed at 6 weeks after tumor cell inoculation. The tumor tissues were excised, paraffin-embedded, formalin-fixed, and the tissue sections (5-mm thick) were made for immunohistochemical staining as described elsewhere.

### Western blot analysis

The cells were lysed with RIPA lysis buffer (Beyotime, Beijing, China) supplemented with a protease inhibitor cocktail (Roche, CA, USA). The cell lysates were separated by 10% SDS-polyacrylamide gel electrophoresis (SDS-PAGE), transferred to 0.22 μm PVDF membranes (Millipore) and incubated with the primary antibodies against cyclin D1, Bcl2, caspase-3, PARP, Slug, Twist, E-cadherin, and N-cadherin (CST) at 4 °C overnight. After incubation with the goat anti-rabbit or anti-mouse secondary antibodies (CST) at 37 °C for 1h, the protein bands were visualized by using chemiluminescence (Millipore). GAPDH was used as loading control.

### Luciferase reporter assay

DANCR promoter was cloned into the pGL3-Basic vector by proof-reading PCR. For the luciferase reporter assay, MGC-803 cells were co-transfected with DANCR promoter luciferase reporter and SALL4 plasmid or SALL4 siRNA as indicated. The Renilla luciferase reporter was used as internal control. The activities of firefly luciferase and Renilla luciferase were quantified by using the dual luciferase reporter assay system (Promega, Madison, WI, USA).

### Chromatin immunoprecipitation assay

The chromatin immunoprecipitation assay was performed in MGC-803 cells by using a commercial kit (Millipore, Darmstadt, Germany) as described previously. After cross-linking, the cells were harvested and the DNA was shredded by sonication. The pre-cleared chromatin was incubated with the antibodies against SALL4 or non-specific IgG overnight. Protein G-agarose beads were added and incubated at 4 °C for 1 h. After reversing the cross-links, the DNA was isolated and used for PCR. The specific primers for PCR detection of the responsive element in DANCR promoter were shown in Supplementary Table 4.

### Statistical analysis

Statistical analysis was performed by using the SPSS 22.0 software (Chicago, IL, USA) and GraphPad Prism 5.0 (La Jolla, CA, USA). Differences between experimental groups were assessed by the Student's *t* test or one-way ANOVA. The associations between DANCR and the clinicopathological features were analyzed by the Pearson χ^2^ test. ROC curve was established to evaluate the diagnostic value of DANCR. All values are expressed as the mean ± SD. All statistical analysis were set with a significance level of *P* < 0.05.

## SUPPLEMENTARY MATERIALS TABLES



## Abbreviations

AUC: area under the ROC curve
CA199: carbohydrate antigen 19-9
CEA: carcinoembryonic antigen
CI: confidence interval
DANCR: anti-differentiation noncoding RNA
EMT: epithelial-mesenchymal transition
EV: extracellular vesicles
GC: gastric cancer
LncRNAs: long noncoding RNAs
RKIP: raf kinase inhibitory protein
ROC: receiver operating curve
SMSCs: synovium-derived mesenchymal stem cells

## References

[1] Choi YJ, Kim N (2016). Gastric cancer and family history. Korean J Intern Med.

[2] Ding Y, Yang Q, Wang B, Ye G, Tong X (2016). The correlation of MGMT promoter methylation and clinicopathological features in gastric cancer: a systematic review and meta-analysis. PLoS One.

[3] Sekar D, Krishnan R, Thirugnanasambantham K, Rajasekaran B, Islam VI, Sekar P (2016). Significance of microRNA 21 in gastric cancer. Clin Res Hepatol Gastroenterol.

[4] Chandra Gupta S, Nandan Tripathi Y (2017). Potential of long non-coding RNAs in cancer patients: From biomarkers to therapeutic targets. Int J Cancer.

[5] Si X, Zang R, Zhang E, Liu Y, Shi X, Zhang E, Shao L, Li A, Yang N, Han X, Pan B, Zhang Z, Sun L (2016). LncRNA H19 confers chemoresistance in ERalpha-positive breast cancer through epigenetic silencing of the pro-apoptotic gene BIK. Oncotarget.

[6] Wang F, Yang H, Deng Z, Su Y, Fang Q, Yin Z (2016). HOX antisense lincRNA HOXA-AS2 promotes tumorigenesis of hepatocellular carcinoma. Cell Physiol Biochem.

[7] Qi F, Liu X, Wu H, Yu X, Wei C, Huang X, Ji G, Nie F, Wang K (2017). Long noncoding AGAP2-AS1 is activated by SP1 and promotes cell proliferation and invasion in gastric cancer. J Hematol Oncol.

[8] Yang G, Lu X, Yuan L (2014). LncRNA: a link between RNA and cancer. Biochim Biophys Acta.

[9] Pan Y, Li C, Chen J, Zhang K, Chu X, Wang R, Chen L (2016). The emerging roles of long Noncoding RNA ROR (lincRNA-ROR) and its possible mechanisms in human cancers. Cell Physiol Biochem.

[10] Ye C, Shen Z, Wang B, Li Y, Li T, Yang Y, Jiang K, Ye Y, Wang S (2016). A novel long non-coding RNA lnc-GNAT1–1 is low expressed in colorectal cancer and acts as a tumor suppressor through regulating RKIP-NF-kappaB-Snail circuit. J Exp Clin Cancer Res.

[11] Zhao QS, Li L, Zhang L, Meng XW, Li LL, Ge XF, Li ZP (2016). Over-expression of lncRNA SBF2-AS1 is associated with advanced tumor progression and poor prognosis in patients with non-small cell lung cancer. Eur Rev Med Pharmacol Sci.

[12] Lv J, Qiu M, Xia W, Liu C, Xu Y, Wang J, Leng X, Huang S, Zhu R, Zhao M, Ji F, Xu L, Xu K (2016). High expression of long non-coding RNA SBF2-AS1 promotes proliferation in non-small cell lung cancer. J Exp Clin Cancer Res.

[13] Xia T, Chen S, Jiang Z, Shao Y, Jiang X, Li P, Xiao B, Guo J (2015). Long noncoding RNA FER1L4 suppresses cancer cell growth by acting as a competing endogenous RNA and regulating PTEN expression. Sci Rep.

[14] Huang M, Hou J, Wang Y, Xie M, Wei C, Nie F, Wang Z, Sun M (2017). Long noncoding RNA LINC00673 is activated by SP1 and exerts oncogenic properties by interacting with LSD1 and EZH2 in gastric cancer. Mol Ther.

[15] Chen WM, Huang MD, Sun DP, Kong R, Xu TP, Xia R, Zhang EB, Shu YQ (2016). Long intergenic non-coding RNA 00152 promotes tumor cell cycle progression by binding to EZH2 and repressing p15 and p21 in gastric cancer. Oncotarget.

[16] Zhou J, Zhi X, Wang L, Wang W, Li Z, Tang J, Wang J, Zhang Q, Xu Z (2015). Linc00152 promotes proliferation in gastric cancer through the EGFR-dependent pathway. J Exp Clin Cancer Res.

[17] Zhao J, Liu Y, Zhang W, Zhou Z, Wu J, Cui P, Zhang Y, Huang G (2015). Long non-coding RNA Linc00152 is involved in cell cycle arrest, apoptosis, epithelial to mesenchymal transition, cell migration and invasion in gastric cancer. Cell Cycle.

[18] Liu YW, Xia R, Lu K, Xie M, Yang F, Sun M, De W Wang C, Ji G (2017). LincRNA FEZF1-AS1 represses p21 expression to promote gastric cancer proliferation through LSD1-Mediated H3K4me2 demethylation. Mol Cancer.

[19] Kretz M, Webster DE, Flockhart RJ, Lee CS, Zehnder A, Lopez-Pajares V, Qu K, Zheng GX, Chow J, Kim GE, Rinn JL, Chang HY, Siprashvili Z (2012). Suppression of progenitor differentiation requires the long noncoding RNA ANCR. Genes Dev.

[20] Chen L, Song Z, Huang S, Wang R, Qin W, Guo J, Lin Z (2016). lncRNA DANCR suppresses odontoblast-like differentiation of human dental pulp cells by inhibiting wnt/beta-catenin pathway. Cell Tissue Res.

[21] Yuan SX, Wang J, Yang F, Tao QF, Zhang J, Wang LL, Yang Y, Liu H, Wang ZG, Xu QG, Fan J, Liu L, Sun SH (2016). Long noncoding RNA DANCR increases stemness features of hepatocellular carcinoma by derepression of CTNNB1. Hepatology.

[22] Jia J, Li F, Tang XS, Xu S, Gao Y, Shi Q, Guo W, Wang X, He D, Guo P (2016). Long noncoding RNA DANCR promotes invasion of prostate cancer through epigenetically silencing expression of TIMP2/3. Oncotarget.

[23] Liu Y, Zhang M, Liang L, Li J, Chen YX (2015). Over-expression of lncRNA DANCR is associated with advanced tumor progression and poor prognosis in patients with colorectal cancer. Int J Clin Exp Pathol.

[24] Zhang L, Xu Z, Xu X, Zhang B, Wu H, Wang M, Zhang X, Yang T, Cai J, Yan Y, Mao F, Zhu W, Shao Q (2014). SALL4, a novel marker for human gastric carcinogenesis and metastasis. Oncogene.

[25] Ma X, Wang X, Yang C, Wang Z, Han B, Wu L, Zhuang L (2016). DANCR acts as a diagnostic biomarker and promotes tumor growth and metastasis in hepatocellular carcinoma. Anticancer Res.

[26] Yang Y, Zhao L, Lei L, Lau WB, Lau B, Yang Q, Le X Yang H, Wang C, Luo Z, Xuan Y, Chen Y, Deng X (2017). LncRNAs: the bridge linking RNA and colorectal cancer. Oncotarget.

[27] Fujimoto A, Furuta M, Totoki Y, Tsunoda T, Kato M, Shiraishi Y, Tanaka H, Taniguchi H, Kawakami Y, Ueno M, Gotoh K, Ariizumi S, Wardell CP (2016). Whole-genome mutational landscape and characterization of noncoding and structural mutations in liver cancer. Nat Genet.

[28] Sun W, Yang Y, Xu C, Xie Y, Guo J (2016). Roles of long noncoding RNAs in gastric cancer and their clinical applications. J Cancer Res Clin Oncol.

[29] Hao YP, Qiu JH, Zhang DB, Yu CG (2017). Long non-coding RNA DANCR, a prognostic indicator, promotes cell growth and tumorigenicity in gastric cancer. Tumour Biol.

[30] Hu HB, Jie HY, Zheng XX (2016). Three circulating lncRNA predict early progress of esophageal squamous cell carcinoma. Cell Physiol Biochem.

[31] Pritchard CC, Kroh E, Wood B, Arroyo JD, Dougherty KJ, Miyaji MM, Tait JF, Tewari M (2012). Blood cell origin of circulating microRNAs: a cautionary note for cancer biomarker studies. Cancer Prev Res (Phila).

[32] Jin C, Shi W, Wang F, Shen X, Qi J, Cong H, Yuan J, Shi L, Zhu B, Luo X, Zhang Y, Ju S (2016). Long non-coding RNA HULC as a novel serum biomarker for diagnosis and prognosis prediction of gastric cancer. Oncotarget.

[33] Karlsson O, Rodosthenous RS, Jara C, Brennan KJ, Wright RO, Baccarelli AA, Wright RJ (2016). Detection of long non-coding RNAs in human breastmilk extracellular vesicles: Implications for early child development. Epigenetics.

[34] Tong X, Gu PC, Xu SZ, Lin XJ (2015). Long non-coding RNA-DANCR in human circulating monocytes: a potential biomarker associated with postmenopausal osteoporosis. Biosci Biotechnol Biochem.

[35] Voutsadakis IA (2016). Epithelial-mesenchymal transition (EMT) and regulation of EMT factors by steroid nuclear receptors in breast cancer: a review and in silico investigation. J Clin Med.

[36] Voutsadakis IA (2012). The ubiquitin-proteasome system and signal transduction pathways regulating Epithelial Mesenchymal transition of cancer. J Biomed Sci.

[37] Voutsadakis IA (2012). Ubiquitination and the ubiquitin-proteasome system as regulators of transcription and transcription factors in epithelial mesenchymal transition of cancer. Tumour Biol.

[38] Zeisberg M, Neilson EG (2009). Biomarkers for epithelial-mesenchymal transitions. J Clin Invest.

[39] Kim T, Jeon YJ, Cui R, Lee JH, Peng Y, Kim SH, Tili E, Alder H, Croce CM (2015). Role of MYC-regulated long noncoding RNAs in cell cycle regulation and tumorigenesis. J Natl Cancer Inst.

[40] Zhang L, Chen S, Bao N, Yang C, Ti Y, Zhou L, Zhao J (2015). Sox4 enhances chondrogenic differentiation and proliferation of human synovium-derived stem cell via activation of long noncoding RNA DANCR. J Mol Histol.

[41] Zhang X, Yuan X, Zhu W, Qian H, Xu W (2015). SALL4: an emerging cancer biomarker and target. Cancer Lett.

[42] Yuan X, Zhang X, Zhang W, Liang W, Zhang P, Shi H, Zhang B, Shao M, Yan Y, Qian H, Xu W (2016). SALL4 promotes gastric cancer progression through activating CD44 expression. Oncogenesis.

[43] Jiang N, Wang X, Xie X, Liao Y, Liu N, Liu J, Miao N, Shen J, Peng T (2017). lncRNA DANCR promotes tumor progression and cancer stemness features in osteosarcoma by upregulating AXL via miR-33a-5p inhibition. Cancer Lett.

[44] Clevers H, Nusse R (2012). Wnt/β-catenin signaling and disease. Cell.

[45] White BD, Chien AJ, Dawson DW (2012). Dysregulation of Wnt/β-catenin signaling in gastrointestinal cancers. Gastroenterology.

