# Supplementary material for: Long noncoding RNA DANCR is activated by SALL4 and promotes the proliferation and invasion of gastric cancer cells
Source: Oncotarget. 2017 Dec 6;9(2):1915–30. doi: 10.18632/oncotarget.23019 (PMC5788609; doi:10.18632/oncotarget.23019)
Supplement: Supplementary file 1 [file oncotarget-09-1915-s001.pdf]

Long noncoding RNA DANCR is activated by SALL4 and promotes the proliferation and invasion of gastric cancer cells

SUPPLEMENTARY MATERIALS

Suppplementary Table 1: The sequences of control and DANCR shRNAs

	Sequence
sh-DANCR	Sense:GATCCGGAGCTAGAGCAGTGACAATGTTCAAGAGACATTGTCACTGCTCTAGCTCCTTTTTC<br>Antisense:AATTGAAAAAAGGAGCTAGAGCAGTGACAATGTCTCTTGAACATTGTCACTGCTCTAGCTCCG
sh-Ctrl	Sense: GATCCGGAGCTCATGGGTCCTTTGTATCGGTACCGATACAAAGGAC CCATGAGCTTTTTTG<br>Antisense: AATTCAAAAAAGCTCATGGGTCCTTTGTATCGGTACCGATACAA AGGACCCATGAGCTCCG

Supplementary Table 2: The sequences of SALL4 and β-catenin siRNAs

	Sequence
si-Control	F: 5'- UUCUCCGAACGUGUCACGUTT -3′<br>R: 5'-ACGUGACACGUUCGGAGAATT-3′
si-SALL4	F: 5'- GUCUCUGGAUGCCUUGAAATT-3′<br>R: 5'-UUUCAAGGCAUCCAGAGACTT-3′
si-β-catenin	F: 5'-CCCAAGCUUUAGUAAAUAUTT-3′<br>R: 5'-AUAUUUACUAAAGCUUGGGTT-3′

Supplementary Table 3: The sequences of primers for qRT-PCR

Gene	Sequence(5′-3′)	Product Size (bp)	Annealing Temperature(°C)
U6	F:5′-CTCGCTTCGGCAGCACA-3′	94	55
	R:5′-AACGCTTCACGAATTTGCGT-3′		
DANCR	F:5′-GCGCCACTATGTAGCGGGTT-3′	96	55
	R:5′-TCAATGGCTTGTGCCTGTAGTT-3′		
E-cadherin	F:5′-CGCATTGCCACATACACTCT-3′	252	55
	R:5′-TTGGCTGAGGATGGTGTAAG-3′		
N-cadherin	F:5′-AGTCAACTGCAACCGTGTCT-3′	337	55
	R:5′-AGCGTTCCTGTTCCACTCAT-3′		
Slug	F:5′-CCTGGTTGCTTCAAGGACAC-3′	395	55
	R:5′-TCCATGCTCTTGCAGCTCTC-3′		
Snail	F:5′-GCGAGCTGCAGGACTCTAAT-3′	310	55
	R:5′-GCCTCCAAGGAAGAGACTGA-3′		
Twist	F:5′-ACGAGCTGGACTCCAAGATG-3′	484	55
	R:5′-GGCACGACCTCTTGAGAATG-3′		
ZEB1	F:5′-CAGAAGCCAGTGGTCATGAT-3′	247	55
	R:5′-GACTGCGTCACATGTCTTTG-3′		
Bax	F:5′-CACCAGCTCTGAGCAGATCAT-3′	214	55
	R:5′-GATCAGTTCCGGCACCTTG-3′		
Bcl-2	F:5′-GGATCCAGGATAACGGAGGC-3′	150	55
	R: 5′-CCAGATAGGCACCCAGGGT-3′		
Cyclin D1	F: 5′-CCGAGAAGCTGTGCATCTAC-3′	221	55
	R: 5′-CTTCACATCTGTGGCACAGAG-3′		

Supplementary Table 4: The sequences of primers for ChIP-PCR

Gene	Sequence(5′-3′)	Product Size (bp)	Annealing Temperature(°C)
ChIP-P1	F:5′- CAACACCTGGGGAAAGGCTA-3′	316	58
	R:5′- GGAGGTTGAGAAGGCAACCA-3′		
ChIP-P2	F:5′- AACTCCTACCCTCCAGCTGAT-3′	256	58
	R:5′- GCATCGAGGTTACTCCAGGG-3′		
ChIP-P3	F: 5′- CCAACCCTGGAGTAACCTCG-3′	308	58
	R: 5′- GTCGGGTAGCTCAGCCAATC-3′		
